# On Affectation in Medicine

**Published:** 1807-09

**Authors:** 


### To Dr. BATTY.

Sir,

MEN of liberal education, I perceive, are ready either to blame or lament every little variety of action or mode of expression, that is not exactly conformable to the settled conduct of their own professional circles; perhaps in essential points, the feeling is proper; yet where this variety does not arise from the *study* to be different, from the *affectation* of eccentricity, these *petty tumours* in society, these little *warts and pimples* in manners are worthy to be as little noticed by the public, as by the individual to whom they belong. But if the individual once begins to be proud of his tumors, and fancies his warts and pimples are beautiful, or the means of acquiring the smallest notoriety, he is then fair game for the Critic, and his *affectation* is just as contemptible as any other species of this ridiculous genus. Now, my dear Doctor, I am not petulant enough to suppose, that your recommendation of seriousness in style, nor the friendly and gentleman-like letter of Dr. Smith, (which deserves my sincere thanks) were levelled at any habitual and inveterate disorder in my manner of expression, but merely to tell me I should observe Hamlet's directions, "suit the words to the action;" that diseases should be detailed in serious language; that physic is a grave grave profession, and its practitioners "sad men." As I am sure these were your motives, I take them in good part, and though I do not conceive it necessary to array a short letter, containing a case, in studied technical phraseology, or in cautious well rounded periods, or pompous verbosity, (these being I should suppose more adapted for essays or orations) yet a letter may occasionally (I speak with submission to your better judgment) be colloquial; it may be as nearly resembling the spontaneous conversation of the person as possible. However, be this as it may, I shall observe especial care to relate the first case that occurs, which I may think worthy the public and your notice, *with all the seriousness the subject demands.*

I know it is imagined by some, that since the canes, ruffles, cloaks, monstrous perriwigs, and other paraphernalia of the Faculty, have given place to a dress and deportment more conformable to the times, that physic and physicians have lost some of their respect from the great world, and nearly the whole of their former estimation and consequence with the vulgar. In my opinion, although I have no wish to "strip medicine of its plumes," this is a *very* vulgar error. If we revert to times when these ornaments were not in fashion, we find the Bishops of former days were not less reverenced in their simple garb, nor the physicians less valued, in dresses far from ostentatious. In after times, the dress, affected deportment, and phraseology of the faculty, arose to such a height of absurdity, that nothing less than the keen satire of MOLIERE could have subdued the monster; and it is certain, more good, more reformation arose in the appearance, education, and conduct of the faculty from the sharpness of his wit, from the ridiculous light in which he displayed their ignorance, follies, and affectation, than from any book of medical ethicks ever published. It must be an agreeable reflection to the faculty to remark, that at present, medical learning and professional skill are justly valued, and obtain their appropriate rank amongst the other learned professions, and are as highly esteemed by the fashionable world, although they are ushered in without the solemn and ridiculous appendages, so essentially necessary seventy years ago.—Not but I think I have observed among some of the faculty of these days, attempts to entrap the world by *affectations* of one sort or other.

The catalogues of the Royal Colleges of Physicians and Surgeons, and the Worshipful Company of Apothecaries, contain no small collection of antiques and curiosities. We

We may see the importance attached to some Gentlemens' wigs; with what solemnity they tap their snuff boxes, and examine their stop-watches. You need not be so ready to start! and tell me there is no harm in this. I say there is harm, they are tricks *ad captandum*. The same remark applies to those who are so pertinaciously technical; these gentlemen discover such *vast constriction of the cuticular capillaries*, and order *deobstruents to promote a diaphoresis, without deranging the catenated associations, &c. &c.* Others affect extreme interest for a patient at the *first interview*; nay, such interest, that undoubtedly they must be the most charming men in the world. No less silly and affected are certain chemists, who blow their filthy *hydrogen* in the face of all companies, and sometimes succeed by their noise and bluster in taking a *weak* part of the town by storm. Now, Sir, these may be all "honourable men," and I am persuaded would be natural men, if they did not conceive they *had parts to act*; they know that the *cullibility* of human nature is infinite, and they wickedly pamper the glutton, instead of endeavouring to restore the constitution of the public, and enlighten the world they live in. As I despise *affectation* in whatever dress it appears, I am as much, nay, more disgusted, with the slovenly, unshaven, uncombed, vulgarity of character certain *Gentlemen* assume: I know it is assumed, their education and writings being in direct opposition to their manners. These men glory in their sneers at all good breeding and gentility, and are almost as offensive as the pretended sanctity and humility observable in some others. When the vulgar detect these tricks, that men of education stoop to practice, I fear it will furnish the strongest arguments for inferior understandings to dispense with believing or practising any thing. I could give you more examples of *affectation*, as combined with some feats of hypocritical dexterity, not at all honourable to some of the profession, but I have no wish to encroach on the pages of your excellent Journal with subjects of this nature. I am more delighted with contemplating the fair side of the picture, and it gives me infinite pleasure in knowing that medical men have now almost universally good educations. For my own part, I reverence the profession, and (with all their foibles) the professors. I believe them, as a *body*, the most learned, and best portion of the public. I know that more private charity is done by them, than by any other class whatever. I know that, in order to gain a livelihood, they have to struggle with more difficulties than in any other profession sion or occupation. I also know they are worse rewarded, and that fewer fortunes are made by them than in any common trade or merchandize.

Having said this in their behalf, I trust you will believe me incapable "of setting down aught in malice;" and as you have been kind enough not to apply *caustic* to my *warts*, I am civil enough to send you no *drastic* in return.

I am, &c.

